# Extended Damage Detection and Identification in Aircraft Structure Based on Multifrequency Eddy Current Method and Mutual Image Similarity Assessment

**DOI:** 10.3390/ma14164452

**Published:** 2021-08-09

**Authors:** Tomasz Chady, Krzysztof Okarma, Robert Mikołajczyk, Michał Dziendzikowski, Piotr Synaszko, Krzysztof Dragan

**Affiliations:** 1 Faculty of Electrical Engineering, West Pomeranian University of Technology in Szczecin, 70-313 Szczecin, Poland; okarma@zut.edu.pl (K.O.); robertmikolajczyk93@gmail.com (R.M.); 2 Airworthiness Division, Air Force Institute of Technology, 01-494 Warsaw, Poland; michal.dziendzikowski@itwl.pl (M.D.); piotr.synaszko@itwl.pl (P.S.); krzysztof.dragan@itwl.pl (K.D.)

**Keywords:** non-destructive testing, aerospace testing, multifrequency and spectrogram eddy current testing, machine vision, image quality

## Abstract

In this paper, a novel approach to Non-Destructive Testing (NDT) of defective materials for the aircraft industry is proposed, which utilizes an approach based on multifrequency and spectrogram eddy current method combined with an image analysis method previously applied for general-purpose full-reference image quality assessment (FR IQA). The proposed defect identification method is based on the use of the modified SSIM4 image quality metric. The developed method was thoroughly tested for various locations, sizes, and configurations of defects in the examined structure. Its application makes it possible to not only determine the presence of cracks but also estimate their size.

## 1. Introduction

Structure diagnostics in terms of damage presence determination is a vital factor in aircraft maintenance systems. At present, most of the used diagnostic techniques include mainly visual inspections and those using Non-Destructive Testing–NDT [1]. Such methods are a part of damage tolerance or fail-safe methodology [2], which assumes the possible existence of failure mode. Each method used for the detection should be adequately selected and tailored to the specified failure mode, including estimating the possibility of detecting damage size [3].

The main phenomena that may lead to catastrophic aircraft damage are corrosion, stress corrosion cracking, and fatigue cycle cracking [4]. In most cases, aerial structures deal with the following damages: fatigue cracking under rivet head in riveted joints, multiple site damage which may lead to widespread fatigue damage, hidden corrosion, cracking, and corrosion in multilayer joints, as well as cracking phenomena due to stress and corrosion [4]. For damage detection of fatigue cracking phenomena, the eddy current method is predominant [5]. The use of eddy current is related to aluminum alloy electric properties, allowing for tests with this method. Moreover, cracking damage geometry is perpendicular to generated eddy current paths in a material that makes the detection easier due to the changes in the electric impedance of the detection unit [6].

Testing with the eddy current method has a comparative character and requires calibration. It constitutes a significant limitation, especially in diagnosing complex geometry elements and multilayer ones [7]. On the other hand, the development of numerical simulation methods allows for creating models that make it possible to determine the distribution of electromagnetic fields and to calculate eddy current characteristics, which may, in turn, make an additional reference to improve the diagnostic process [7,8].

According to the industry survey of structural health monitoring technology and NDT usage [9], crack detection is one of the most essential and current aerospace industry needs in implementing the NDT and Structural Health Monitoring (SHM) systems. The most problematic are inspections of multilayer riveted structures for detecting fatigue cracks propagating from the rivet hole, especially in lower layers [10]. For such structures, the crack size and depth determination are crucial issues from the detection accuracy point of view.

As Mottl proposed using the Dodd and Deeds model [11], detection parameters may be determined theoretically for the selected probe type and geometry. However, due to the local variations of the conductivity, surface treatment, and specific types of probes, as Mook et al. indicate [12], the current density may differ from the theoretical ones, which implies the design of probes.

Several types of probes can be used for damage position, size, and orientation determination: manual (reflection, absolute, differential), rotating and sliding [13].

Several constraints in selecting the proper inspection approach should be considered, such as time of inspection, geometrical implications, surface treatment conditions, the possibility of damage detection, and sensitivity [14,15].

Therefore, an application of the NDT methods for materials used in the aircraft industry is one of the most demanding areas of research in the era of Industry 4.0, integrating the knowledge of electromagnetic measurements, particularly the eddy current method, with data analysis methods originating from signal and image processing domain.

In many cases, the results of non-destructive measurements take the form of 2D or 3D images, and therefore image analysis methods are of particular interest. Regardless of the potential use of image quality assessment (IQA) methods for an automatic evaluation of the usefulness of individual images or measurements for further analysis, some of the IQA metrics might also be applied as tools for detecting the presence of material defects or even for their classification.

Unfortunately, the applicability of many full-reference (FR) metrics, considered the most popular and the most universal, may be limited by assuming the necessary knowledge of the reference image, which may be unavailable in many practical applications [16,17,18,19]. Another limitation is the prerequisite perfect spatial adjustment and identical size (in terms of pixel resolution) of both compared images, which may require additional image registration and cropping. Therefore, a potential application of the FR IQA metrics for NDT purposes would require their modifications, as proposed in this paper, where the idea of the mutual similarity evaluation is considered.

The rest of the paper is organized as follows: In Section 2, the inspection approach is presented (Section 2.1), followed by discussing measurements (Section 2.2) and the discussion of similarity-based IQA methods (Section 2.3). The proposed method of image analysis is described in Section 2.4, and the discussion of the obtained results is provided in Section 3, followed by concluding remarks.

## 2. Materials and Methods

### 2.1. Approach to Inspection

An example of non-destructive technologies for structural integrity monitoring of the aerial components considered in this paper is the eddy current inspection performance on the PZL-130 Orlik aircraft structure. Within the conducted Full-Scale Fatigue Test (FSFT) for that aircraft, a program of detailed NDT inspections was scheduled. A part of that program was the Eddy Current inspection delivery, especially for critical parts of the wing structure. Inspection delivered during the FSFT revealed the existence and development of more than 100 failure modes, which were detected and described.

According to the best practice standards, the exemplary failure modes that can be found during the aircraft inspection are as follows [20]: hole peripheral cracks, through-thickness cracks in radius, hole through-thickness cracks, and hole radial countersink cracks.

**Figure 1:**
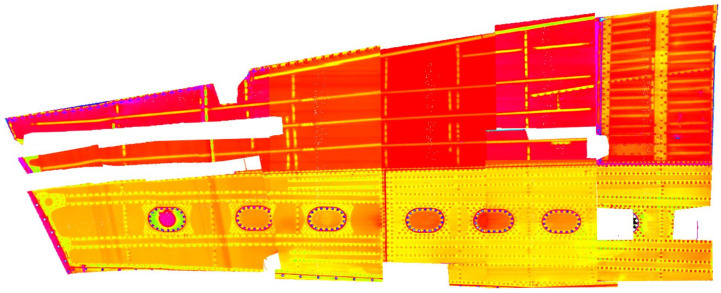
Eddy current scan of the wing structure.

Several eddy current techniques were implemented for damage detection, such as high-frequency reflection probes, high- and low-frequency automated scanning, and the magneto-optic imaging MOI™ system for the general and detailed structure visualization. Figure 1 presents the example of the wing structure automated inspection result using the MAUS^®^ mobile system and C-scan data presentation. Such presentation mode shows the signal value (impedance module) as a function of the sensor location.

The inspection was delivered with the 20 kHz for surface-breaking cracks and 1 kHz for the subsurface, consecutively. Scan resolution (pixel-to-pixel size) was equal to 1 mm. Based on collected data, the NDT operator provides data analysis using visual recognition of the anomaly, which may exist on the provided image. That analysis is being supported by signal classification and description tools embedded in the system. Moreover, the data export function enables more advanced analysis based on signal processing methods [21].

Such an inspection method has also benefited from data comparison possibility, a valuable function for the FSFT to monitor damage nucleation and damage progression. A more detailed analysis is possible for specific regions of interest (ROI) with the complex impedance plane analysis, signal evaluation, and size quantification tool.

**Figure 2:**
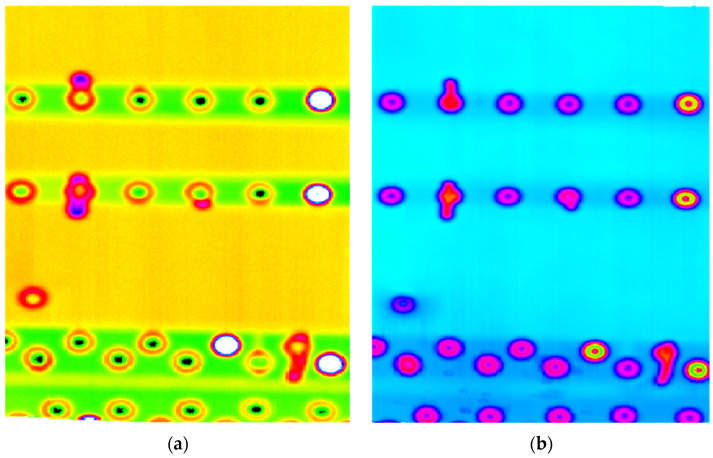
Eddy current scan of the region of interest obtained for the 20 kHz excitation signal: (**a**) imaginary part; (**b**) real part.

Figure 2 presents the result of the failure presented as the hole radial countersink crack. The combined presentation of the real and imaginary part of the impedance allows evaluating damage severity and creates an opportunity to evaluate crack orientation and depth of location. However, with the lower frequency inspection, damage presence is not as clear as with higher ones and may cause some difficulties in recognizing deeper located flaws.

Once the FSFT inspection was completed, the need for an increase in the damage detection sensitivity, especially in the second or third layer, has arisen.

The approach to the detection and classification of damage considered in this article is based on using a probe adapted to detect damage in multilayer aluminum aircraft structures, an advanced multifrequency inspection method, and the mutual image similarity assessment.

From the observations and practical inspection, the use of a dedicated probe with the proper signal-to-noise ratio and efficient penetration depth is a required tool for detailed aircraft inspection. In the case of structures with multiple layers, it is also crucial to utilize many frequencies.

Analysis of the various signals achieved from all the frequencies is a complicated and time-consuming procedure. For this reason, the automation of the defect detection and identification process is essential for the proper and effective functioning of the inspection system.

In the classical ECT systems, the use of several frequencies requires the application of several probes with valid central frequencies to obtain the estimated depth of penetration. Additional limitations also come from a decrease in the current density with an increase in depth of penetration. Such phenomena reduce inspection reliability and affect the probability of damage detection. Thus, the inspection of multilayer structures and geometry constraints requires specialized probes and signal processing to keep linearity of damage detection and thickness change. In the proposed multifrequency ECT system, many of these limitations were overcome.

### 2.2. Data Acquisition

In the eddy current method, the measurement sensitivity depends on several factors, such as the transducer dimensions, an excitation frequency, and properties of a tested structure (permeability and conductivity). Unfortunately, high sensitivity and a high resolution usually cannot be achieved at the same time. Enlargement of the probe dimensions will result in higher sensitivity (for deeply loaded defects) and a lower spatial resolution. Decreasing the excitation frequency will cause a similar effect. In testing multilayer aircraft structures, high and possibly similar sensitivity for detecting defects in different layers and at different depths is required. At the same time, it is necessary to maintain the appropriate spatial resolution. It is crucial to detect even the most minor cracks in the vicinity of holes after riveted connections. In this case, it is particularly advantageous to use a measuring system based on the Massive Multifrequency Excitation and Spectrogram Method (MMFES), proposed in [22]. This method uses a complex signal containing many sinusoidal components as an excitation signal and a spectrogram to detect and identify defects. A large variety of frequency components creates an opportunity for all defects to be detected using the most appropriate testing frequency. It is for the first time to apply the MMFES method for the inspection of riveted structures.

#### 2.2.1. Measuring System Description

The measurements were carried out using the computer system implementing the Massive Multi-Frequency Excitation and Spectrogram (MMFES) method. In the MMFES system, a complex waveform is used as the excitation, created by adding sinusoidal components with precisely defined frequencies and amplitudes. The formula which describes the function defining the excitation voltage is as follows:

(1) $$s_{E}\left(t\right)=\overset{n}{\underset{i=1}{\sum }}U_{i}\cdot \sin\left(2\pi f_{i}\;t+\phi_{i}\right).$$

where *s_E_*–waveform of the excitation signal, *n*–number of harmonics, *U_i_*–amplitude of the *i*-th component, *f_i_*–amplitude of the *i*-th component, *ϕ_i_*–phase of the *i*-th component.

**Figure 3:**
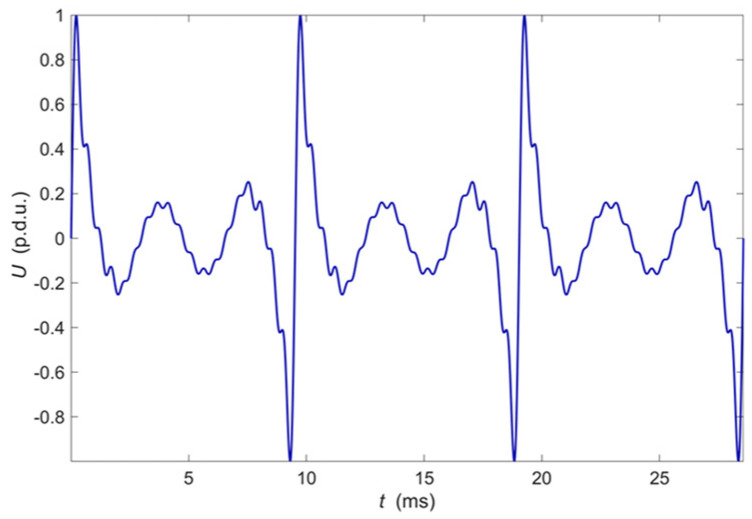
The plot of excitation voltage measured on the output of the D/A converter.

**Figure 4:**
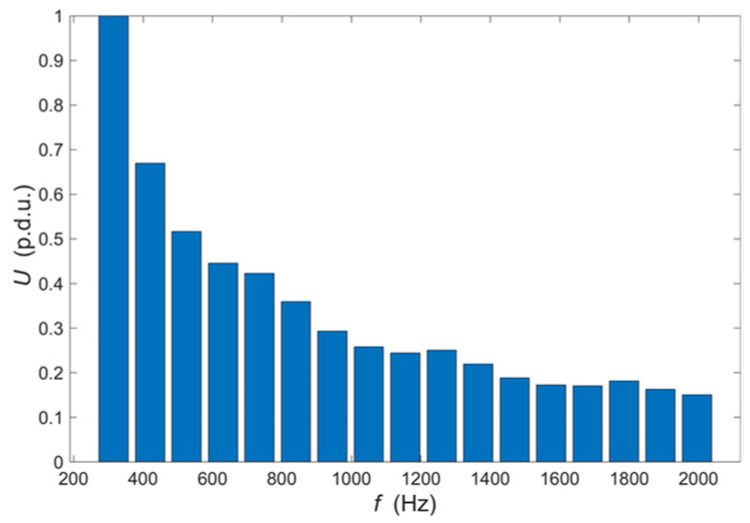
The amplitudes of the harmonics used to synthesize excitation voltage waveform shown in Figure 3.

The frequency range is selected in such a way as to include the values for which the maximum interaction of eddy currents with defects occurs at possible depths of the tested structure. The number of harmonic components *n* is selected in such a way as to achieve the highest possible signal to noise ratio at the eddy current transducer output. The relation of individual harmonics amplitude to the expected noise level and the possibility of filtering signals in the frequency domain are considered. The initial phases of individual harmonics do not significantly affect the ability to detect defects, but it is possible to limit the maximum value achieved by the excitation signal by appropriate selection. One of the critical issues is the selection of the excitation harmonic amplitudes. They are experimentally determined so that the harmonic components of the signal measured at the transducer output have the same value when it is located over the tested material that does not contain heterogeneity. The amplitude equalization process usually requires two to three iterations until the differences are satisfactorily reduced (i.e., below 1%). The experimental process allows eliminating the influence of the transducer frequency characteristic, lead wires, and other factors such as a lift-off and the inclination of the transducer concerning the tested material surface. The plot of excitation voltage measured on the output of the D/A converter as a function of time is shown in Figure 3. The amplitude spectrum of this voltage is shown in Figure 4.

The excitation driving voltage is generated in the D/A converter (NI PXI 5422-sample rate 200 MS/s, 80 MHz bandwidth, 16-Bit waveform generator) and then fed through the high-frequency power amplifier (NF HAS 4101, frequency range DC to 10 MHz, slew rate 5000 V/μs, max. current 1.4 A, amplification gain 20) to the excitation coils of the eddy current transducer.

**Figure 5:**
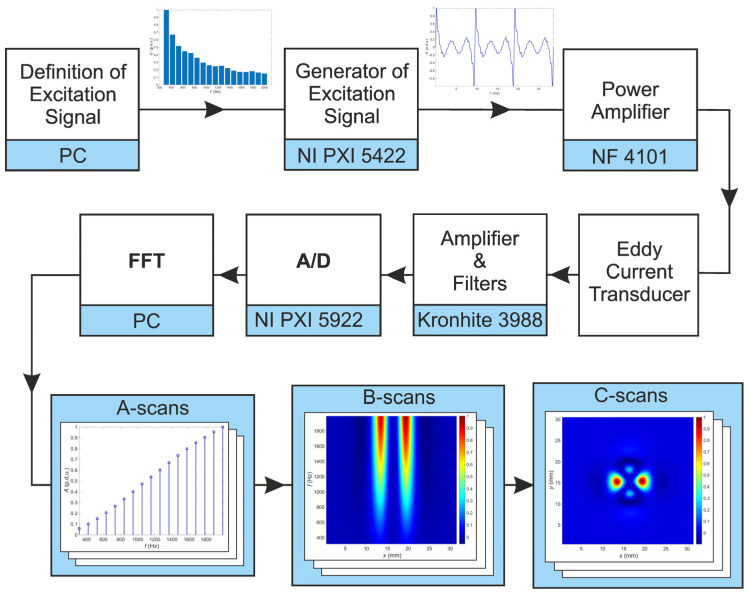
The block scheme of the MMFES system.

The voltage generated in the measuring coil of the eddy current transducer is amplified and filtered with the Kronhite 3988 and then digitized in the NI PXI 5922 A/D converter (maximum sampling rate 15 MS/s, maximum resolution 24 bits). Due to the high resolution and accuracy of the A/D converter, which is achieved while maintaining a relatively high conversion rate, it is possible to obtain precise information about minor changes of amplitudes of the harmonic components in the processed signals. In these measurements, the sampling rate was set to 315 kS/s, which allows a resolution of 24 bits to be achieved. One of the critical issues in the MMFES system is to minimize spectral leakage. Therefore, the sampling frequency is precisely selected, considering the harmonic frequencies present in the excitation signal. Additionally, a clock that controls the A/D converter is used simultaneously to control the D/A converter generating the excitation. The block scheme of the MMFES system is shown in Figure 5. The digitized signal from the sensing coil is then processed using the Fast Fourier Transform (FFT) to determine the individual harmonic amplitudes. The values of harmonic amplitudes acquired for a single transducer position constitute the frequency characteristic of the transducer and the material within the effective range of the generated electromagnetic field.

**Figure 6:**
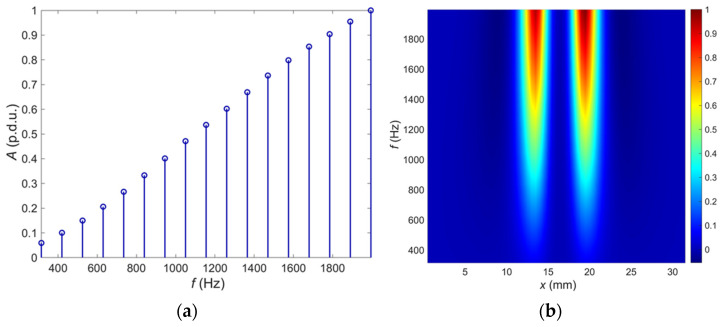
An example of: (**a**) the A-scan–frequency characteristics, position of the sensor *x* = 19.5 mm, *y* = 15.5 mm; (**b**) B-scan–spectrogram, position of the sensor *y* = 15.5 mm.

Signals obtained in the Ultrasonic Testing (UT) are commonly displayed in three different formats. The formats are known in the NDT terminology as A-scan, B-scan, and C-scan presentations. Each presentation format provides different information about the region of material being inspected. The A-scan presents the amount of received ultrasonic energy as a function of time for a single position of the transducer. Peaks visible in the A-scan presentation allow to estimate the location and size of the discontinuity in the inspected material. Similar information can be achieved from the frequency characteristic obtained in the MMFES ECT system. Therefore, we will use the term A-scan for such kind of data presentation despite some significant differences. An example of the MMFES ECT A-scan in the 300 Hz–2 kHz range is shown in Figure 6.

In the UT method, the image composed of A-scans captured during the transducer’s linear scanning is called a B-scan. From the B-scan, it is possible to identify the depth of the discontinuity and its linear dimension in the scanning direction. A C-scan is an image obtained during two-dimensional scanning (*x*, *y*) of the transducer and showing values for a selected time of acquisition. Finally, D-scan is a three-dimensional representation of the signals measured during (*x*, *y*) scanning.

Moving the eddy current probe in a line over the tested material and registering the frequency characteristics for the following positions enables creating a spectrogram. The spectrogram is a two-dimensional plot of the relative amplitude of the signal frequency components from the pick-up coil versus the transducer *x* position. The spectrogram amplitude can be calculated as a difference between the current amplitude and the amplitude measured over the uniform material to emphasize material structure changes. The spectrogram corresponds to a B-scan.

**Figure 7:**
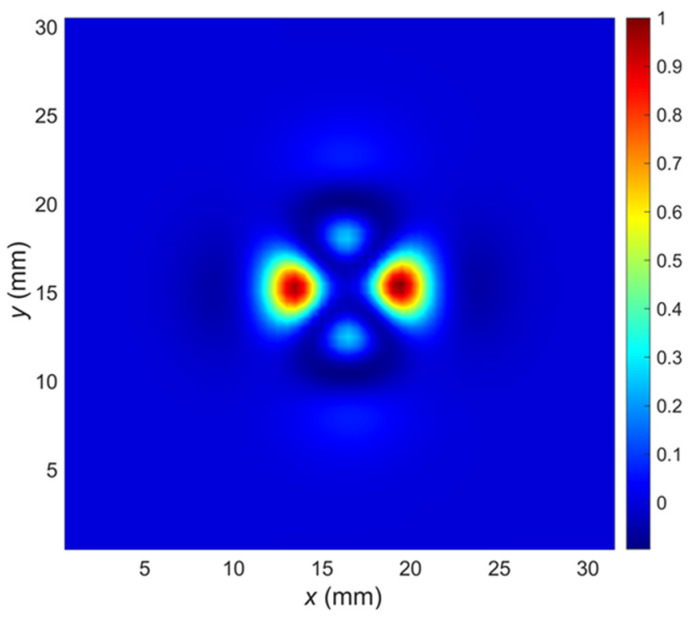
An example of the C-scan (2D plot of the values vs. sensor coordinates, excitation frequency *f* = 1995 Hz).

Successive scanning of the tested element, line by line, allows creating a two-dimensional plot of the signal value against the orthogonal scanning directions (*x* and *y*). The plot of the signal value for a single frequency as a function of the transducer *x* and *y* positions (Figure 7) is the equivalent of a C-scan.

Finally, the signal value three-dimensional plot against frequency *f* and the transducer position coordinates (*x*, *y*) corresponds to a D-scan.

The C-scans taken for the selected frequencies will be the input images to the automatic defect recognition system using image quality assessment based on similarity.

#### 2.2.2. The Eddy Current Transducer

**Figure 8:**
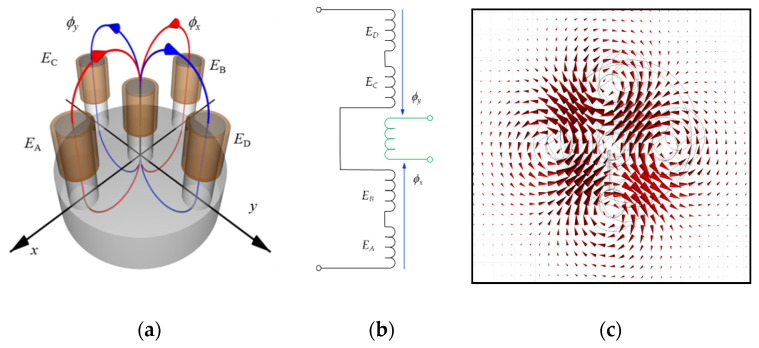
The eddy current transducer: (**a**) the schematic view; (**b**) the electric circuit; (**c**) the distribution of calculated eddy currents induced in the specimen.

Inspection of the aluminum multilayer structures required a transducer characterized by high sensitivity, deep penetration, and the ability to detect defects in all directions with similar sensitivity. The eddy current differential transducer (Figure 8a) consists of a ferrite core with five symmetrically placed columns.

A receiving coil containing 100 turns is wound on the central column. Four excitation coils (*E_A_*, *E_B_*, *E_C_*, *E_D_*) with 25 turns each are wound on the remaining columns. The excitation coils (*E_A_* and *E_B_*) are connected in series, as are the *E_C_* and *E_D_* coils (Figure 8b). Both pairs are generating in the pick-up coil opposite directed magnetic fluxes. The resulting flux in the pick-up coil is close to zero in the equilibrium state. The output signal depends on a difference of fluxes *ϕ_x_* and *ϕ_y_*. The differential configuration of the transducer enables easy detection of the lack of symmetry in the tested specimen. Figure 8c shows the distribution of eddy currents induced in the specimen obtained by numerical analysis of the transducer.

Although dedicated for tube inspection, the transducer with a quite similar configuration was presented for the first time [23].

**Figure 9:**
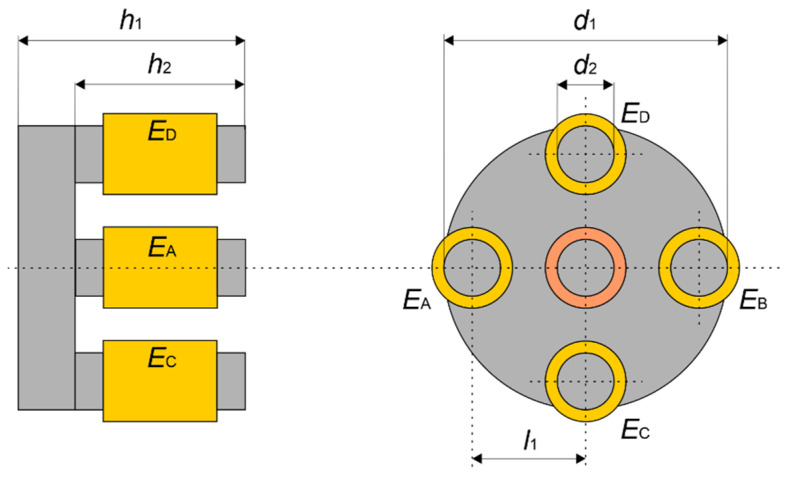
Dimensions of the eddy current transducer: *d*_1_ = 12 mm, *d*_2_ = 2 mm, *l*_1_ = 5 mm, *h*_1_ = 10 mm, and *h*_2_ = 6 mm.

The transducer size was selected, considering the distribution of eddy currents induced in the tested specimen and the necessity to detect defects located in the deepest layers of the structure. The core dimensions are provided in Figure 9.

#### 2.2.3. Test Samples and Measurements

**Figure 10:**
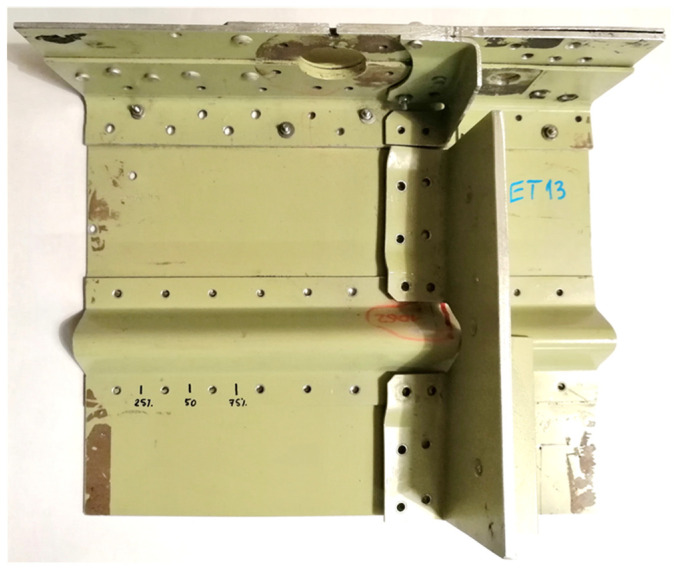
Photo of the airplane sample used in the initial experiments.

Preliminary experiments were carried out on a specimen (Figure 10) taken from an aircraft operated under various load spectra. High loads initiated crack propagation at the edges of the rivet holes.

**Figure 11:**
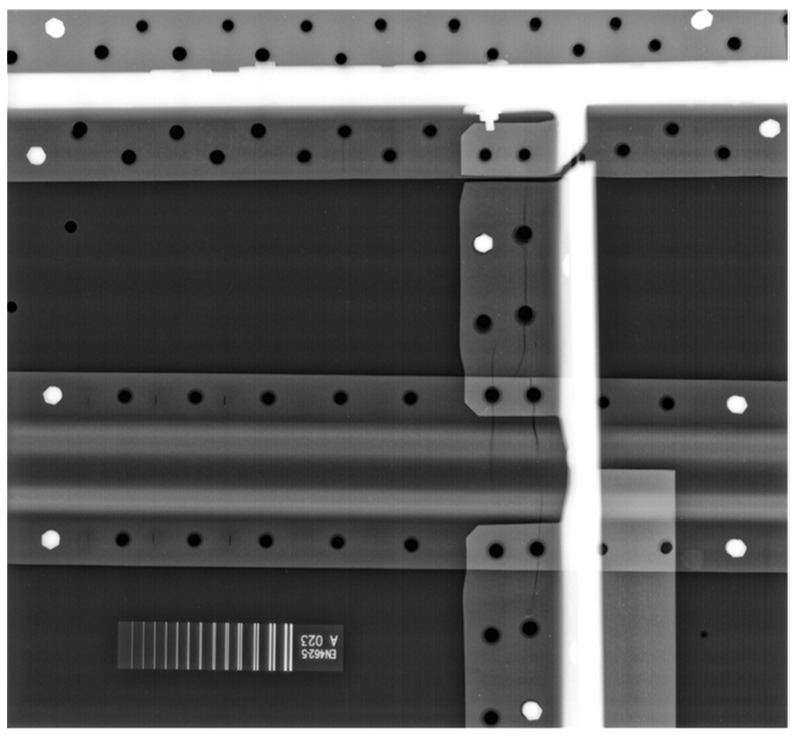
X-ray image of the airplane sample used in the initial experiments.

The cracks are very well visible in the X-ray image (Figure 11) of the sample. Most of the observed cracks occurred in the first layer of the examined structure. Only some of the cracks appeared in the second layer (deeper under the surface). All the cracks were propagated from the edges of the rivet holes. The vast majority of the cracks were directed in one of two perpendicular directions due to the load’s directivity.

Most of these cracks were of considerable length (longer than 5 mm). There were also long cracks in the second layer, but it was observed that some of the cracks were less than 2 mm long.

This research aimed to develop a universal identification algorithm that works appropriately with defects of various sizes and orientations. However, as mentioned earlier, aircraft specimens contained a relatively limited set of naturally formed defects. For this reason, it was decided to produce aluminum specimens with a similar structure but with defects of a much more different nature, both in configuration and length.

**Figure 12:**
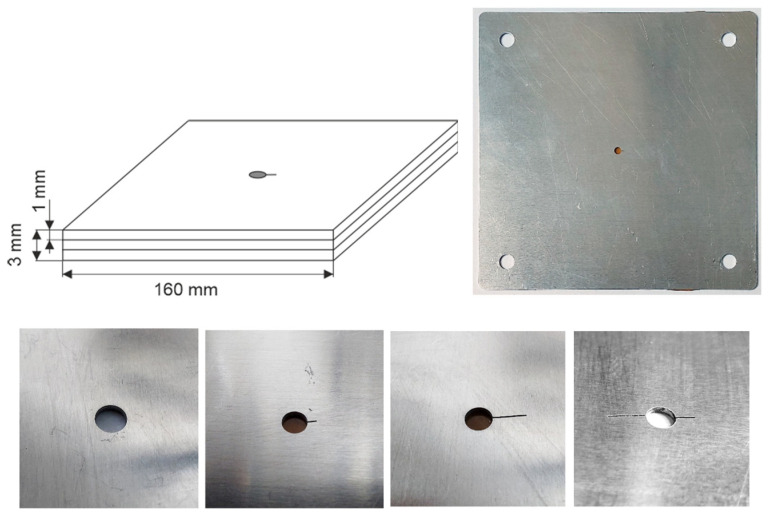
The aluminum test specimens used in the experiments.

Each sample consists of three 1 mm thick aluminum alloy plates joined together (Figure 12). The test plates were sufficiently large (160 mm × 160 mm) that measurements could be made without significant influence of the edges. In all the plates, the holes were made in the center using a CNC machine. The diameter of the holes was 4 mm. In several plates, cuts simulating cracks of various lengths (2 mm, 5 mm, and combined two cuts 2 and 5 mm) were manufactured, as shown in Figure 12.

In the research, we used 40 different samples, which were created from the assembly of the presented four plates with artificial defects in various configurations (defects in one plate and several plates simultaneously, on different layers, rotated by different angles, etc.). The use of various variants of the arrangement of basic defects creates a chance to test the system in a fairly wide range. However, it is planned to further expand the database of samples with artificial defects to verify responses in less frequent situations. At the same time, work is underway to collect a set of samples taken from real objects, but unfortunately, the number of experimental samples is limited by their availability. The defects found there are not very diverse and most often have larger dimensions than those that we would like to detect during routine inspections. For this reason, it is crucial to operating in two ways, collecting actual samples and using samples with artificially generated defects.

The measurements were performed by scanning the probe over the area surrounding the hole in steps of 0.5 mm in *x* and *y*-direction. The measurements were made using the multifrequency excitation signal consisting of sinusoidal components with frequencies from 315 Hz up to 1995 Hz (17 frequency components). The lift-off (the gap between the specimen and the sensor) was measured to be 0.4 mm.

**Figure 13:**
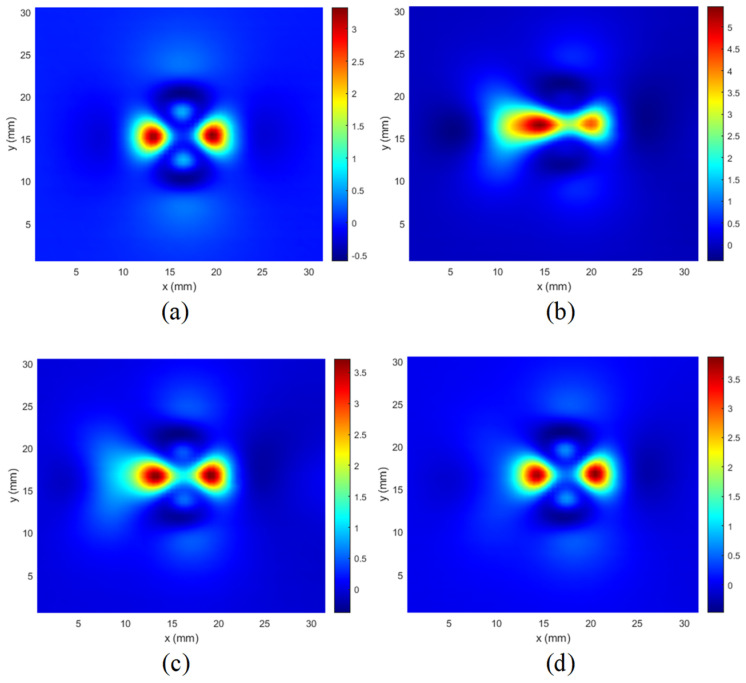
Comparison of C-scans: (**a**) achieved in case of the specimen without defect; (**b**) the specimens with the 5 mm long crack along the *x*-axis in the 1st layer; (**c**) in the 2nd layer; (**d**) in the 3rd layer.

**Figure 14:**
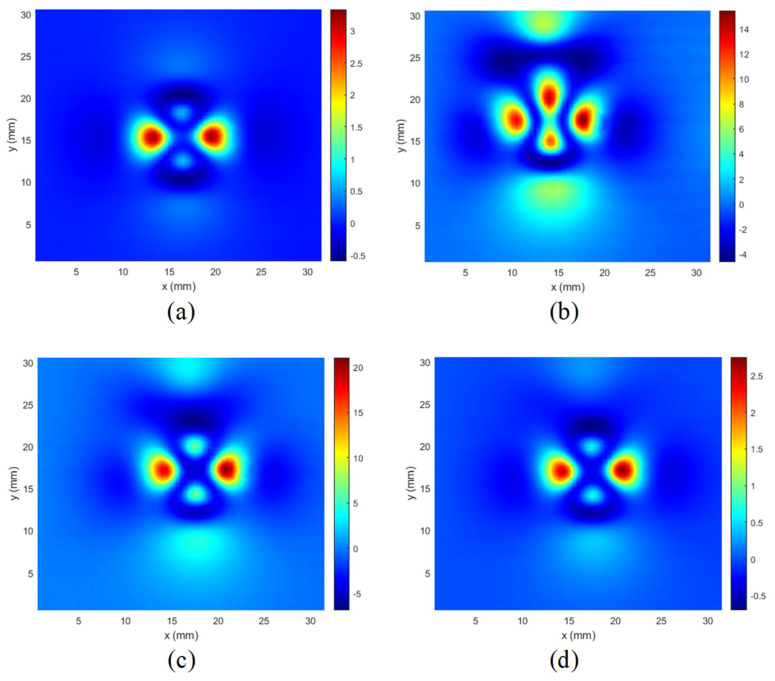
Comparison of C-scans: (**a**) achieved in case of the specimen without defect; (**b**) the specimens with the 5 mm long crack along the *y*-axis in the 1st layer; (**c**) in the 2nd layer; (**d**) in the 3rd layer.

**Figure 15:**
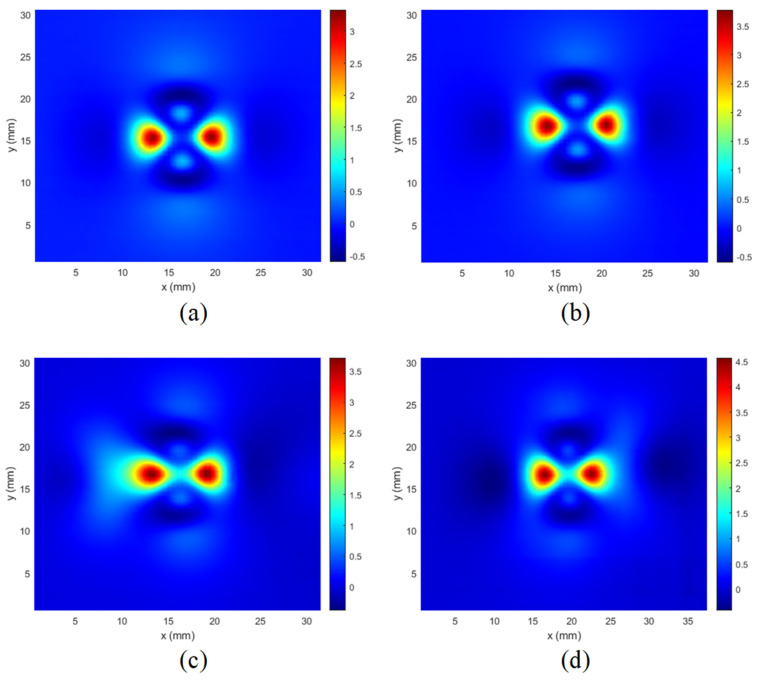
Comparison of C-scans: (**a**) achieved in case of the specimen without defect; (**b**) the specimens with the crack in the 1st layer, along the *x*-axis, and with a length of 2 mm; (**c**) length 5 mm; (**d**) combined two cracks with a length of 2 mm and 5 mm.

The resulting C-scans for the lowest excitation frequency are presented in Figure 13, Figure 14 and Figure 15. In Figure 13, the results of measurements made for a hole with defects (5 mm long crack) on different layers are presented. It can be seen that the C-scan measured for the rivet hole without defects (Figure 13a) is very symmetrical, and the presence of a defect on any layer causes a significant disturbance of the symmetry of the response (Figure 13 b–d).

A similar phenomenon can be observed that it occurs even more intensively in the case of cuts manufactured at an angle of 90 degrees along the *y* axis (Figure 14).

However, as shown in Figure 15, in the case of minor cuts or when they are deeper below the surface (Figure 13d), the symmetry disturbances are not very large, and for effective detection of defects, it is necessary to use appropriate identification algorithms.

It is worth mentioning that the signals (C-scans) achieved for the other frequencies were not significantly different. Therefore, only one frequency component was selected for the presentation.

### 2.3. Similarity-Based Image Quality Assessment

Examples of the eddy current C-scans for a specified ROI using a single frequency are shown in the previous subsection as color plots. Nevertheless, the type of measurement data obtained as the C-scans is single-channel, similarly as in the case of greyscale images, although the data range is different. Hence, their color representation is unsuitable from the image processing point of view, where various three-channel color spaces are applied to analyze color images. Additionally, most state-of-the-art image quality assessment (IQA) methods, described further, were developed for monochrome images. Therefore, the obtained results were converted into grayscale 2D images, using 8-bit values in the range 0–255, for which the considered IQA methods may be directly applied.

Since the expected features of such obtained images are their symmetry and self-similarity, as it may be observed in Figure 13a, Figure 14a and Figure 15a, the similarity-based IQA metrics are of primary interest in further investigations.

General-purpose image quality assessment methods, including those used in our experiments, may be considered as useful approaches for many applications where the image or video quality plays an important role, e.g., making it possible to skip the degraded frames during the analysis of video sequences or to optimize some newly developed image filtering or lossy compression algorithms.

On the other hand, the universal full-reference IQA methods, based on the comparison of an undistorted reference (“pristine”) image with the degraded one, may also be used for the detection of some types of contamination or image distortions as well as the estimation of their amount or level.

Although in many applications, where the reference image might be unavailable, the most desired approach would be the no-reference (NR) IQA, also referred to as “blind”, such methods are usually quite complex, and their performance is lower than state-of-the-art FR IQA metrics. Usually, such NR metrics are sensitive only to a limited number of distortion types, hence their universality is relatively low.

Nevertheless, the development of new IQA methods during recent years [17,18,19,24] has been concentrated on the possibly best correlation of the newly proposed metrics with subjective quality evaluations for the publicly available image datasets containing numerous degraded images, together with Mean Opinion Score (MOS) values gathered from human observers.

The most popular FR metric, known as Structural Similarity (SSIM), was proposed in [25], being the combination of three factors representing the luminance distortions, loss of contrast, and structural distortions. Since the formula of the SSIM utilizes the averaging of the local similarity indexes calculated for each position of the sliding window (by default, 11 × 11 pixels Gaussian window is used), it is possible to build the quality map as well. Nevertheless, in most practical applications, a single scalar index is preferred, possibly well correlated with human perception of various distortions.

The simplified idea of the calculation of the local SSIM value between the blocks *x* and *y*, representing two fragments of the compared images for the specified location of the sliding window, may be expressed as the product of three components:

(2) $$SSIM=\frac{2\cdot \mu_{x}\cdot \mu_{y}+C_{1}}{\mu_{x}^{2}+\mu_{y}^{2}+C_{1}}\cdot \frac{2\cdot \sigma_{x}\cdot \sigma_{y}+C_{2}}{\sigma_{x}^{2}+\sigma_{y}^{2}+C_{2}}\cdot \frac{\sigma_{xy}+C_{3}}{\sigma_{x}\cdot \sigma_{y}+C_{3}},$$

where $\mu_{x}$ and $\mu_{y}$ are the mean values whereas $\sigma_{x}$ and $\sigma_{y}$ denote the variance values in blocks *x* and *y*, respectively, and $\sigma_{xy}$ stands for the covariance. Three stability constants: $C_{1}={\left(0.01\cdot L\right)}^{2}$, $C_{2}={\left(0.03\cdot L\right)}^{2}$ and $C_{3}=0.5\cdot C_{2}$, prevent the possible division by zero for very dark or flat areas of the image, assuming that *L* is the dynamic range of pixels values. The final SSIM result is obtained as the average of local values calculated for all window positions.

Following the first success of the SSIM, many similar approaches and extensions of this metric were proposed by various researchers during recent years, starting from its multi-scale version, known as MS-SSIM [26], making its results independent of the sliding window size. Some other modifications of the idea of Structural Similarity utilize the calculations of gradients, leading to Gradient Similarity [27] as well as Gradient Magnitude Similarity Deviation (GMSD) [28]. Another direction of development of SSIM-inspired metrics is related to the use of Riesz transform [29,30] and phase congruency, leading to well-performing Feature Similarity (FSIM) [31], where the similarity of gradient magnitude is combined with the similarity of the phase congruency, calculated similarly as in the SSIM formula.

The same general approach is also used in the Edge Strength Similarity metric [32], as well as in some other metrics utilizing local variance [33] or spectral residual [34]. Another interesting approach, originating from the idea of the SSIM, is based on the perceptual combination of multi-scale gradient magnitude maps that concern the micro- and macrostructural similarity that are further combined with color information similarity and high-distortion-based pooling, forming the final Perceptual Similarity (PSIM) metric [35].

Nevertheless, although the performance of most of these improved metrics is significantly better in comparison to the “original” SSIM for many IQA benchmarking datasets, their application for some other types of images, e.g., containing multiple distortions [36] or for the surface quality assessment of 3D prints [37] not always leads to satisfactory results. Some other examples might be the development of quality metrics for audio-visual signals, stitched images [38], or light field reconstruction, compression, and display [39], where some specific types of distortions may take place. Therefore, the application of the general-purpose IQA methods in some other areas is not always straightforward and may require their extensions, significant modifications, and an appropriate choice of potentially useful metrics.

**Figure 16:**
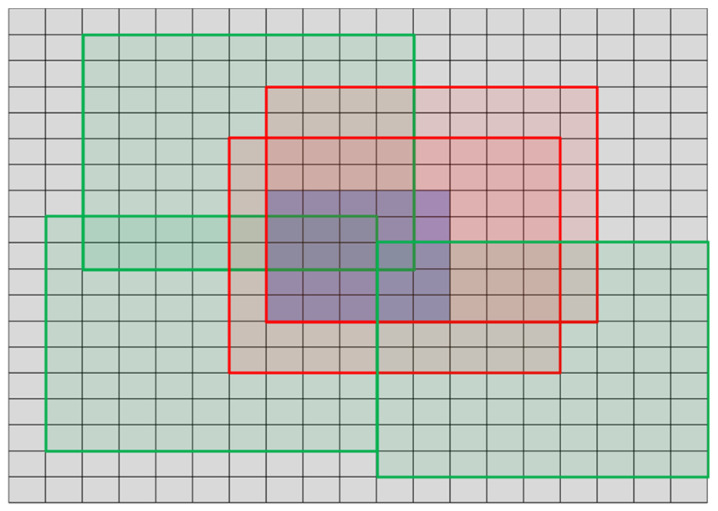
Illustration of the sample blocks included (green) and excluded (red) from the calculations of blocks predictability.

A remarkable extension of the SSIM formula was proposed by Ponomarenko et al. [40], where the fourth multiplicative component was introduced, which is related to the similarity of predictability of image blocks. This component may be determined by calculating the mean square error (MSE) between the specified block and the neighboring blocks. Hence, the minimum MSE value is chosen as the local result and then multiplied by the three “original” SSIM components, namely similarity of the average luminance of image blocks, a correlation factor, and contrast similarity. Nevertheless, the additional necessity of searching for the most similar block increases the computational complexity. The formula of the method denoted as SSIM4 may be expressed as:

(3) $$SSIM4=\frac{2\cdot \mu_{x}\cdot \mu_{y}+C_{1}}{\mu_{x}^{2}+\mu_{y}^{2}+C_{1}}\cdot \frac{2\cdot \sigma_{x}\cdot \sigma_{y}+C_{2}}{\sigma_{x}^{2}+\sigma_{y}^{2}+C_{2}}\cdot \frac{\sigma_{xy}+C_{3}}{\sigma_{x}\cdot \sigma_{y}+C_{3}}\cdot \frac{2\cdot \varepsilon_{x}\cdot \varepsilon_{y}+C_{4}}{\varepsilon_{x}^{2}+\varepsilon_{y}^{2}+C_{4}},$$

where $C_{4}=C_{2}$ is assumed, $\varepsilon_{x}^{2}$ and $\varepsilon_{y}^{2}$ are the measures of predictability of the respective image blocks expressed as mean MSE values between the given block *A* and its neighboring blocks *B* according to

(4) $$\varepsilon_{A}^{2}=\underset{B}{\min}\left(\frac{1}{M\cdot N}\cdot \sum_{i=1}^{M}\overset{N}{\underset{j=1}{\sum }}{\left(A_{ij}-B_{ij}\right)}^{2}\right),$$

assuming that neighboring blocks *B* are limited by a specified distance (the default parameters are search area 19 × 19 pixels and the block size *M* = *N* = 9). Nevertheless, the blocks that contain the whole central part consisting of 5 × 5 pixels block are excluded from the calculations of the predictability measure $\varepsilon_{A}^{2}$ [33] as illustrated in Figure 16, where three excluded red blocks and three green blocks are shown.

Although all SSIM-based metrics utilize the estimation of image similarity, they cannot be applied directly for the analysis of images obtained as the results of measurements considered in this paper. Hence, their modification related to the calculation of the mutual (internal) similarity between the image fragments is postulated.

The concept of similarity calculation between two images without using reference images was also used in blind image quality assessment, although in this case, the use of the pseudo-reference image (PRI) is necessary [41,42] is based on the use of multiple PRIs, assuming various degrees of distortion aggravation.

Nevertheless, the approach proposed in this paper is different since it does not require any comparisons with any other images, and the only required data for the mutual similarity calculation is the image that is the result of eddy current measurements.

### 2.4. The Proposed Image Analysis Method

#### 2.4.1. Symmetry Detection

As the samples without any defects should provide symmetrical images obtained as measurements, the first necessary task is to detect the origin as the measurement results are not necessarily centered on the image plane. Unfortunately, considering the influence of defects and the presence of the measurement noise, the application of moment-based methods, e.g., the Centre of Gravity, has not led to satisfactory results. Nevertheless, the proper results for all considered samples, i.e., the detection of an individual pixel representing the center of symmetry of measurement data, were obtained employing the correlation between the original image and its horizontally or vertically flipped equivalents. After determining the locations of the maximum correlation values, the images are shifted and cropped, forming four quadrant images, which may be used as inputs for mutual similarity calculations.

#### 2.4.2. Thresholding and Normalization of Data Range

Before the symmetry detection, the grayscale images were initially normalized to the range 0–255, where 0 corresponds to the lowest measured value, and 255 represents the highest peak value. To decrease the influence of the highest peaks on the obtained results and make it possible to detect the presence of smaller defects, which influence the lower values the additional thresholding and normalization were conducted, equivalent to image saturation, assuming the preservation of only the lower 20% of the original range for simplicity the threshold value equal to 50 was chosen for 8-bit grayscale images). Such obtained images, divided into four quadrants expected to be symmetrical, form the input data for the mutual similarity calculations.

#### 2.4.3. Mutual Image Similarity Calculation

Since most of the FR IQA metrics were proposed assuming that the compared images represent the same scene and only one of these images is corrupted, they might also be applied for comparison purposes, assuming that both images are highly similar. Another essential requirement is the proper registration of both images; hence the precise determination of the center of symmetry is a crucial issue, influencing the obtained results.

**Figure 17:**
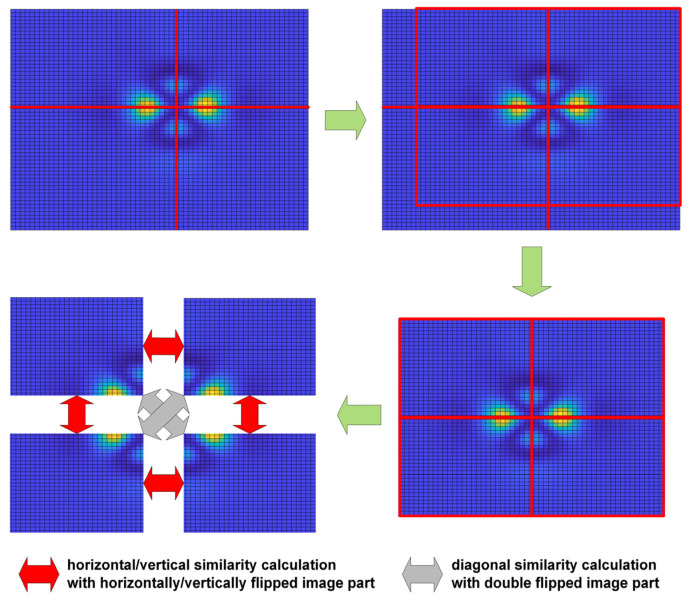
Illustration of the idea of mutual similarity calculation for the symmetrical fragments of the sample image.

Studying the obtained experimentally images, both horizontal and vertical symmetry may be easily observed as expected for the images without defects. Hence, the division of images into four fragments was proposed making it possible to calculate four similarity factors between each part and the flipped neighboring fragment, as shown in Figure 17. For the images where the center of symmetry is shifted from the center of the image, an appropriate cutting of boundary rows or columns should be made to ensure the same size of both compared image fragments.

A similar approach to mutual similarity calculation was also used for the aesthetic quality evaluation of the 3D printed surfaces [37]. However, in this case, a precise alignment has not been necessary, and no symmetry assumptions were utilized. Nonetheless, in the problem investigated in this paper, image flipping should be applied. Nevertheless, the diagonal similarities, i.e., these between the top left and bottom right (or top right and bottom left) fragments, marked by gray arrows in Figure 17, are not necessarily calculated to accelerate the computations.

During the conducted experiments, several similarity-based IQA metrics discussed in Section 2.3 were verified, starting from the “classical” SSIM formula [25], through FSIM [31], PSIM [35], and other modifications, to the SSIM4 metric [40], leading to the most accurate classification of samples, presented further.

Nevertheless, it should be noted that the direct application of the FR IQA would be impossible without the knowledge of the reference images of metal structures without cracks which should be precisely registered with the analyzed images. Therefore, we proposed a novel approach assuming the calculation of the mutual similarities reflecting the expected symmetry of the images obtained using the multifrequency eddy current method. As the typical methods used for this purpose failed in the initial experiments, we adapted some metrics that are well-known and commonly accepted for general-purpose IQA, although the application of the Structural Similarity and the other IQA metrics for the classification of samples required the use of the average and maximum values of the mutual similarities obtained for 17 frequencies, not just the simple calculation of their values.

## 3. Results and Discussion

**Figure 18:**
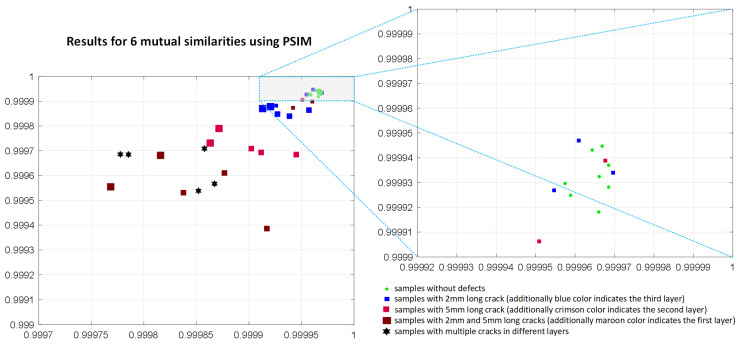
Results obtained applying six mutual comparisons employing the PSIM metric using the maximum and average of its values calculated for 17 images corresponding to various frequencies used in experiments.

**Figure 19:**
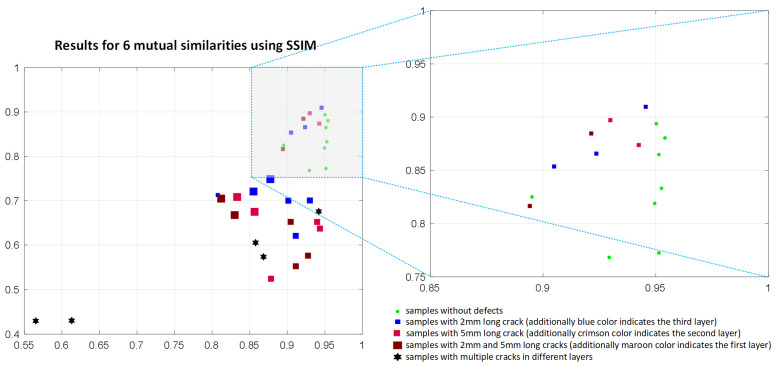
Results obtained applying six mutual comparisons employing the SSIM metric using the maximum and average of its values calculated for 17 images corresponding to various frequencies used in experiments.

**Figure 20:**
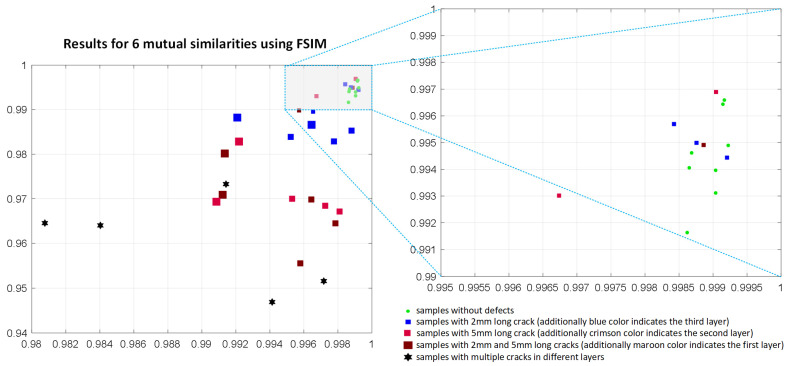
Results obtained applying six mutual comparisons employing the FSIM metric using the maximum and average of its values calculated for 17 images corresponding to various frequencies used in experiments.

During the conducted experiments, several similarity-based IQA methods were analyzed and verified towards their potential application as the similarity measures in the investigated problem. To illustrate the non-applicability of most of the considered metrics the results achieved for the PSIM, assuming the use of six mutual similarities, are presented in Figure 18. The results achieved using the SSIM and FSIM metrics applied for the mutual similarity calculations are illustrated in Figure 19 and Figure 20, respectively. As it may be easily observed, the discrimination between the samples without defects and these containing them is not possible in such cases.

Similarly, the application of some other metrics considered in the paper, except the proposed application of the SSIM4, has not led to satisfactory results, regardless of the analysis of the maximum, minimum, average, or median values of the mutual similarities.

**Figure 21:**
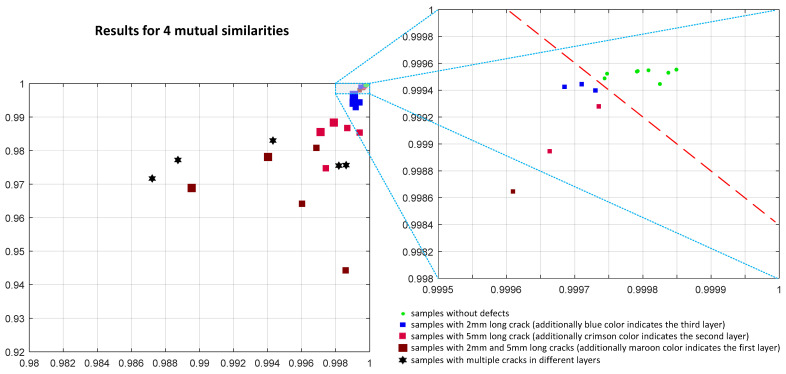
Results obtained applying four mutual comparisons employing the SSIM4 metric using the maximum and average of its values calculated for 17 images corresponding to various frequencies used in experiments.

Much better results, presented in Figure 21, were obtained using four mutual similarities (horizontal and vertical) calculated according to the SSIM4 formula. Analyzing the plots, one may observe that reliable discriminations between the samples without defects and these containing some cracks are possible. It was achieved using a combination of two features, namely the highest values of the average and maximum of four mutual similarities, determined for each of 17 images obtained from measurements conducted using various frequencies (from 315 Hz to 1995 Hz).

**Figure 22:**
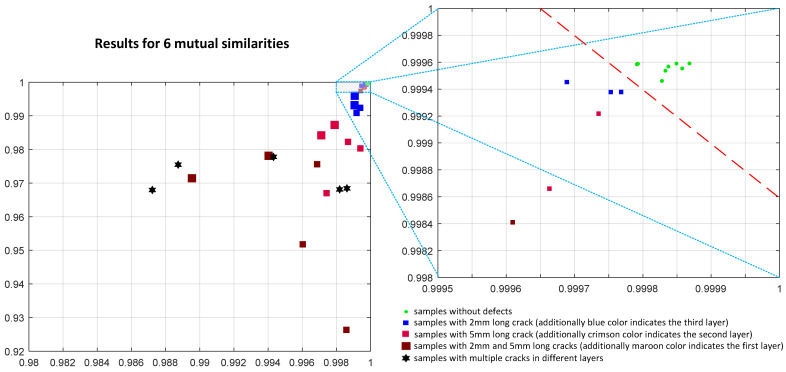
Results obtained applying six mutual comparisons employing the SSIM4 metric using the maximum and average of its values calculated for 17 images corresponding to various frequencies used in experiments.

Even better discrimination may be observed using six mutual similarities, as shown in Figure 22, where an improved separation between the green points representing the samples without cracks and the others may be easily observed. Both plots contain the results of measurements and further analysis conducted for 36 samples, where various colors indicate the depth of cracks and their size corresponds to the size of individual points.

Since some samples used in experiments contain multiple cracks, they were marked with black hexagons. As it may be observed, there is a relation between the size of the crack (as well as its depth) and the values of both considered features, as well.

Nevertheless, correct classification of samples into high-quality ones and those containing some cracks may be conducted using the product of two considered features (as well as their sum), particularly assuming the calculation of six mutual comparisons.

**Figure 23:**
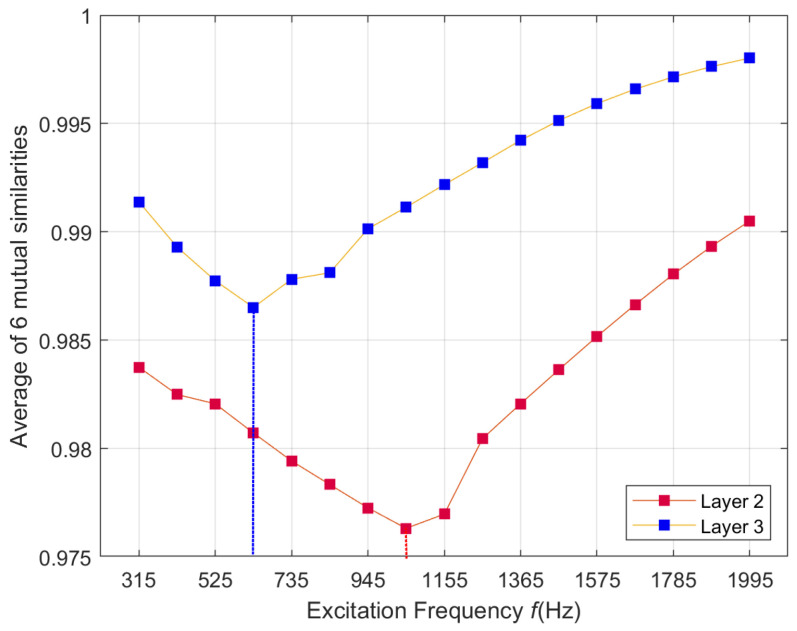
Frequency dependence of the average of mutual similarities calculated using the proposed method.

The applied multifrequency method allows for even more detailed identification of defects. Thanks to the access to information obtained at many frequencies, it is possible to determine the layer of the structure containing the damage more precisely. Figure 23 shows an example of the frequency characteristics of the obtained similarity. It can be observed that the minimum similarity for the defect located in the third layer is achieved at a significantly lower frequency (*f* = 630 Hz) than in the case of the defect located in the second layer (*f* = 1050 Hz). This effect can be further emphasized by using a wider range of excitation frequencies. It is crucial in the case of surface defects where the applied frequencies should be much higher. Nevertheless, due to the limited maximum excitation frequency, the plot of the frequency characteristics for the defect in the first layer did not contain the extreme point. This curve is not shown in Figure 23 due to significantly lower similarity values, which would make the remaining plots unreadable.

The broader range of excitation frequencies (and higher maximum excitation frequency) may cause lower sensitivity to defects occurring in the third layer or below. The provision of the ability to detect deeply located defects has resulted from specialists’ requirements from the aviation industry.

## 4. Concluding Remarks

Currently, the eddy current techniques used for damage detection in the aerospace industry require impedance plane analysis or C-scan data visualization to evaluate damage existence. Each data collected must be evaluated by the qualified operator. Such an approach requires a relatively large amount of time for analysis and data comparison. Moreover, the sensitivity of inspection is decreased with the use of lower frequencies. Therefore, the application of the proposed method is interesting for the further development of an efficient damage detection methodology in the aerospace industry.

The solution proposed in the paper makes it possible to determine the presence of cracks in the aircraft structures using the mutual image similarity analysis of images obtained as the result of the multifrequency eddy current measurements. After validating the usefulness of several image quality assessment metrics, the results achieved by the application of the modified SSIM4 method were encouraging, allowing not only for the detection of structural defects but also for the estimation of their size.

A relatively large number of experiments with defects of various sizes, configurations, and locations in the studied structure were carried out. The possibility of both single cracks and complicated, complex damages with cracks propagating in many directions was considered. Such a complementary set of data made it possible to thoroughly test the proposed solution.

The experimental verification of the proposed approach confirms its potential industrial application, mainly due to a fast calculation of the quality metric compared to the total measurement time. The computation of the final combination of two features based on six mutual similarities using the SSIM4 metric together with visualization takes a few seconds. The calculations of the mutual similarities for different layers and areas may be easily parallelized as well. Assuming that the number of rivets in 1 m^2^ of the specimen is around 150, the analysis using non-optimized Matlab code will take about 15 min. Analysis can partially overlap with the measurement process. The time can be considerably reduced using GPU and parallel computing for data acquisition (FFT analysis) and defect detection. Another aspect is the time needed to perform the measurements. A significant limitation, in this case, is the lowest excitation frequency, as it is necessary to measure at least half of the period. In the case of the excitation frequency as low as 300 Hz, the measurement of 1 m^2^ with a resolution of 0.5 mm will take at least 50 min.

According to our best knowledge, the proposed method is the first attempt to use image quality assessment methods to analyze eddy current measurements, particularly concerning the application of the multifrequency eddy current method to the inspection of riveted structures. In contrast to many popular deep learning approaches, our method is explainable as its main assumption is based on detecting the expected symmetry and its numerical estimation related to the size of the cracks.

## Data Availability

The data presented in this study are available on request from the corresponding author. The data are not publicly available due to a complicated structure that requires additional explanations.
